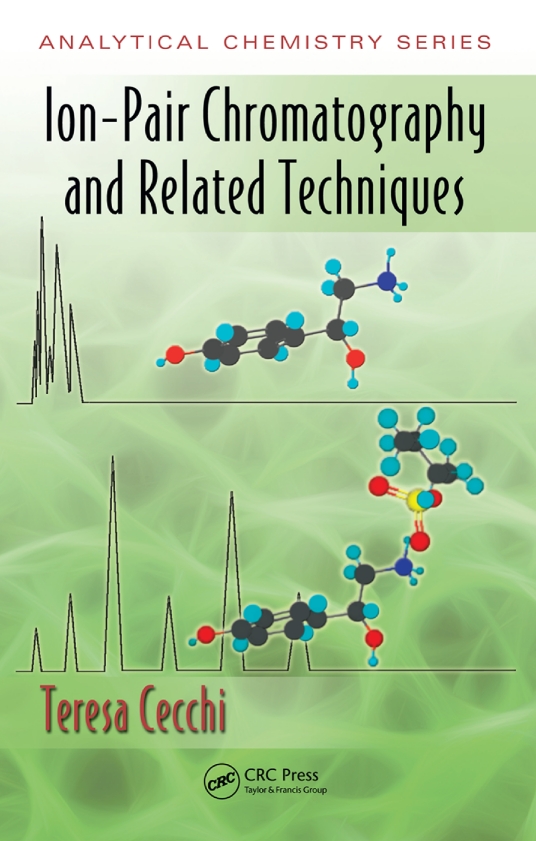# Teresa Cecchi: Ion-pair chromatography and related techniques

**DOI:** 10.1007/s00216-012-5945-3

**Published:** 2012-04-01

**Authors:** Bogusław Buszewski

**Affiliations:** Department of Environmental Chemistry and Bioanalytics, Faculty of Chemistry, Nicolaus Copernicus University, Gagarin St. 7, 87-100 Toruń, Poland

**Book’s topic** Most of the analytes determined by chromatographic techniques are polar or charged ones; therefore, there is a great need to improve final separation results because normal or reversed phase modes are lacking in selectivity. Ion-pair chromatography (IPC) is a great solution for these drawbacks. The subject of the book is therefore very interesting for chromatographers working with polar and charged substances. This book provides a broad coverage of the subject of IPC with regard to theoretical features, the parameters influencing the results and the applications. The reader will find a discussion of all aspects of IPC, beginning with the theoretical basis of this technique and its retention mechanism. The author introduces to the reader all the issues connected with stationary phases, ion-pair reagents, organic modifiers, the pH of the eluent, temperature, and various detection techniques which may be used in IPC. One can also find much practical advice on how to start with IPC and how to improve results by the use of IPC.

**Contents** The book consists of 17 chapters. The first and second chapters describe the history of IPC and the beginnings of this technique. Also discussed are many aspects connected with the thermodynamic basis of ion-pair formation and the techniques used in the study of the ion-pairing phenomenon. The next two chapters deal with different general parameters influencing modelling of the retention mechanism (mobile phase composition and nature of the analyte). Both theoretical and empirical models of IPC are discussed. Chapters 5-7 describe in a complex and detailed manner the parameters important for optimization of the final IPC results. The stationary phases for normal and reversed phase modes, with both conventional and fast IPC columns, are introduced. Most of the traditional, volatile and chaotropic ion-pair reagents are also described. The great advantage of this part of the book is the holistic review of new classes of ion-pair reagents, namely ionic liquids, acyl monoglycinate surfactant, violet dye and 3,5-dinitrobenzoic acid. Moreover, Chap. 8 discusses the influence of organic modifiers on the IPC retention process. The stationary phase coverage with a ion-pair reagent decreases when the organic modifier content is high. Furthermore, the polarity of the organic solvent in the mobile phase is shown to have a great influence on the IPC results. Chapter 9 is devoted to the role of the pH of the eluent and the issues relating to modification of the pH. The next chapter concerns the influence of temperature on column efficiency and selectivity. The special modes of IPC are described in Chap. 11. They are connected with the use of a mixture of various ion-pair reagents, the addition of ion-pair reagents to the sample, the application of special additives and the modification of the ionic strength of the eluent at a constant concentration of the ion-pair reagent. Chapter 12 is of great value because it describes various detection techniques and their coupling with IPC. The basic advantages of IPC and comparisons of its coupling with UV–vis, fluorescence, electrical conductivity, electrochemical, evaporative light scattering, and mass spectrometry detectors are introduced here. Valuable information for the analyst working with various samples is provided in Chap. 13. This chapter presents examples of applications of IPC for different types of samples of various origins, namely inorganic species and food, pharmaceutical, toxicological, clinical and environmental samples. Chapter 14 presents a brief comparison of IPC with other chromatographic techniques, such as ion chromatography, capillary electrophoresis and gas chromatography. The next chapter is a continuation of the previous one. It describes in a brief and concise manner the application of the ion-pairing phenomenon in capillary electrophoresis as well as supercritical fluid chromatography, UV–vis spectrophotometers, extraction and sample preparation. Chapter 16 presents results of non-separative applications of IPC. Here, the utilization of ion pairing in denaturing high-performance liquid chromatography is mainly presented. The last chapter contains concluding remarks and describes future research needs.

**Comparison with existing literature** The book focuses in depth on a theoretical description of IPC and the parameters influencing the separation process. Because theory leads to practice, this book is a valuable counterpart to other publications. It may be of particular interest to readers who are starting to use IPC, but it will also be interesting for those who wish to broaden their knowledge of IPC.

**Critical assessment** The book is a great compendium of the history, basis, theory, practice and application of IPC. It covers a wide range of issues connected with this technique and therefore it is very valuable for analysts working not only with IPC but also with all modes of liquid chromatography. The monograph is well written and the content can be easily understood by chromatographers beginning to work with IPC as well as experienced chromatographers.

**Summary** The monograph *Ion-pair chromatography and related techniques* has excellent value and is a great source of knowledge for scientists and analysts working with IPC. It is a quite well-balanced mixture of theoretical considerations and practice advice for what to do to improve the final result. The book also highlights recent developments in the field of column technology, ion-pair reagents, special ion-pair modes and coupling.